# Methoxo[*N*′-(3-meth­oxy-2-oxidobenzyl­idene)benzohydrazidato]oxidovanadium(V)

**DOI:** 10.1107/S1600536810009608

**Published:** 2010-03-27

**Authors:** Shu-Mei Huang, Fei-Feng Jiang, Xiao-Hua Chen, Qiong-Jie Wu

**Affiliations:** a College of Chemistry and Materials Science, Fujian Normal University, Fuzhou, Fujian 350007, People’s Republic of China; b College of Life Science, Fujian Agriculture and Forestry University, Fuzhou, Fujian 350002, People’s Republic of China

## Abstract

In the title complex, [V(C_15_H_12_N_2_O_4_)(CH_3_O)O], the V^V^ ion exhibits a distorted square-pyramidal coordination geometry; three donor atoms from a hydrazone ligand and one O atom of the deprotonated methanol define the coordination basal plane. The V^V^ ion is displaced by 0.464 (1) Å from the basal plane towards the axial oxide O atom. Intra­molecular O—H⋯N hydrogen bonding occurs. Inter­molecular C—H⋯O hydrogen bonding is also observed in the crystal structure.

## Related literature

For general background to hydrazones and their chelation ability, see: Liu & Gao (1998 ▶); Ma *et al.* (1989 ▶); Sur *et al.* (1993 ▶); Sun *et al.* (2005 ▶). For related structures, see: Chen *et al.* (2004 ▶); Liu *et al.* (2006 ▶); Ghosh *et al.* (2007 ▶); Seena *et al.* (2008 ▶). For the synthesis, see: Gao *et al.* (1998 ▶); Chen (2008 ▶).
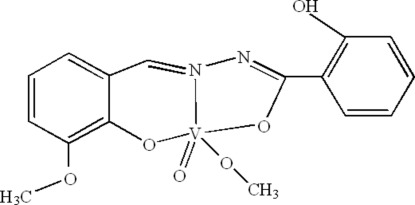


## Experimental

### 

#### Crystal data

[V(C_15_H_12_N_2_O_4_)(CH_3_O)O]
*M* *_r_* = 382.24
Monoclinic, 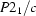

*a* = 16.194 (3) Å
*b* = 6.6746 (13) Å
*c* = 15.359 (3) Å
β = 96.89 (3)°
*V* = 1648.1 (6) Å^3^
*Z* = 4
Mo *K*α radiation
μ = 0.64 mm^−1^
*T* = 293 K
0.39 × 0.22 × 0.15 mm

#### Data collection

Rigaku R-AXIS RAPID diffractometer
Absorption correction: multi-scan (*TEXRAY*; Molecular Structure Corporation, 1999 ▶) *T*_min_ = 0.845, *T*_max_ = 0.909
14918 measured reflections
3686 independent reflections
2776 reflections with *I* > 2σ(*I*)
*R*_int_ = 0.051

#### Refinement

*R*[*F* ^2^ > 2σ(*F* ^2^)] = 0.042
*wR*(*F* ^2^) = 0.118
*S* = 1.08
3686 reflections
228 parameters
H-atom parameters constrained
Δρ_max_ = 0.33 e Å^−3^
Δρ_min_ = −0.39 e Å^−3^

### 

Data collection: *TEXRAY* (Molecular Structure Corporation, 1999 ▶); cell refinement: *TEXRAY*; data reduction: *TEXSAN* (Molecular Structure Corporation, 1999 ▶); program(s) used to solve structure: *SHELXS97* (Sheldrick, 2008 ▶); program(s) used to refine structure: *SHELXL97* (Sheldrick, 2008 ▶); molecular graphics: *ORTEX* (McArdle, 1995 ▶); software used to prepare material for publication: *SHELXL97*.

## Supplementary Material

Crystal structure: contains datablocks I, global. DOI: 10.1107/S1600536810009608/xu2731sup1.cif

Structure factors: contains datablocks I. DOI: 10.1107/S1600536810009608/xu2731Isup2.hkl

Additional supplementary materials:  crystallographic information; 3D view; checkCIF report

## Figures and Tables

**Table 1:** Selected bond lengths (Å)

V1—O1	1.8277 (17)
V1—O3	1.9436 (16)
V1—O5	1.5736 (18)
V1—O6	1.7351 (16)
V1—N1	2.0963 (17)

**Table 2:** Hydrogen-bond geometry (Å, °)

*D*—H⋯*A*	*D*—H	H⋯*A*	*D*⋯*A*	*D*—H⋯*A*
O4—H4*B*⋯N2	0.82	1.84	2.568 (2)	147
C8—H8*A*⋯O4^i^	0.93	2.32	3.243 (3)	169

Symmetry code: (i) 
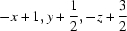
.

**Fig. 1.**
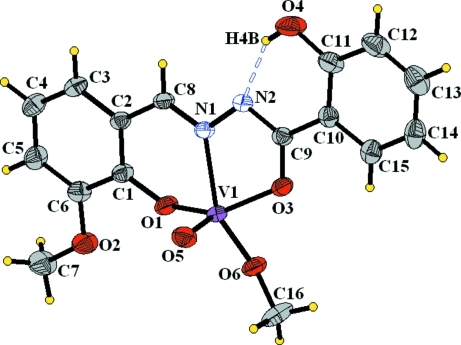
The molecular structure of the title compound, showing 30% probability displacement ellipsoids for non-H atoms. A dashed line indicates the intramoleculat hydrogen bonding.

## supplementary crystallographic information

### Comment

Hydrazones are of interest owing to their capacity for chelating to transition (Sur *et al.*, 1993; Sun, *et al.*, 2005), lanthanide (Ma *et al.*, 1989) and main group (Liu & Gao 1998) metals.

The V^V^ ion exists in a distorted square-pyramidal coordination geometry. Three donor atoms (O1, O3 and N1) of the hydrozone ligand and O6 atom from the methanol group define the coordination basal plane, with a maximum mean plane deviation of 0.0215 (9) Å. The V atom is displaced towards the axial oxo O atom by 0.464 (1)Å from the basal plane. Bond distances (Table 1) and bond angles around V1 atom are compared with those in reported oxovanadium complexes (Chen *et al.*, 2004; Seena *et al.*, 2008; Liu *et al.*,2006; Ghosh *et al.*, 2007). In the crystal structure there are the intramolecular O—H···N hydrogen bonding and intermolecular C—H···O hydrogen bonding (Table 2).

### Experimental

VO(acac)_2_ (acac = acetylacetonate) was synthesized according to the reported method (Gao *et al.*, 1998). The hydrazone ligand (L) was prepared by following a similar procedure reported by Chen (2008).

The title compound was prepared by reacting H_2_L (0.1 mmol) with VO(acac)_2_ (0.1 mmol) in methnol/water solution (10 ml) with stirring. The solution was filtered and allowed to stand at room temperature for one week, dark-red crystals of complex (I) were obtained.

### Refinement

All H atoms were placed in idealized positions and treated as riding with O—H = 0.82 Å, C—H = 0.93-0.96 Å; U_iso_(H) = 1.2U_eq_(C) or 1.5U_eq_(C,O).

### Crystal data

**Table:**

[V(C_15_H_12_N_2_O_4_)(CH_3_O)O]	*F*(000) = 784
*M**_r_* = 382.24	*D*_x_ = 1.541 Mg m^−^^3^
Monoclinic, *P*2_1_/*c*	Mo *K*α radiation, λ = 0.71073 Å
Hall symbol: -P 2ybc	Cell parameters from 2776 reflections
*a* = 16.194 (3) Å	θ = 3.1–27.5°
*b* = 6.6746 (13) Å	µ = 0.64 mm^−^^1^
*c* = 15.359 (3) Å	*T* = 293 K
β = 96.89 (3)°	Prism, dark-red
*V* = 1648.1 (6) Å^3^	0.39 × 0.22 × 0.15 mm
*Z* = 4	

### Data collection

**Table:**

Rigaku R-AXIS RAPID diffractometer	3686 independent reflections
Radiation source: fine-focus sealed tube	2776 reflections with *I* > 2σ(*I*)
graphite	*R*_int_ = 0.051
ω scans	θ_max_ = 27.5°, θ_min_ = 3.1°
Absorption correction: multi-scan (*TEXRAY*; Molecular Structure Corporation, 1999)	*h* = −21→21
*T*_min_ = 0.845, *T*_max_ = 0.909	*k* = −8→8
14918 measured reflections	*l* = −19→19

### Refinement

**Table:**

Refinement on *F*^2^	Primary atom site location: structure-invariant direct methods
Least-squares matrix: full	Secondary atom site location: difference Fourier map
*R*[*F*^2^ > 2σ(*F*^2^)] = 0.042	Hydrogen site location: inferred from neighbouring sites
*wR*(*F*^2^) = 0.118	H-atom parameters constrained
*S* = 1.08	*w* = 1/[σ^2^(*F*_o_^2^) + (0.062*P*)^2^ + 0.2985*P*] where *P* = (*F*_o_^2^ + 2*F*_c_^2^)/3
3686 reflections	(Δ/σ)_max_ = 0.001
228 parameters	Δρ_max_ = 0.33 e Å^−^^3^
0 restraints	Δρ_min_ = −0.39 e Å^−^^3^

### Special details

**Table:**

Geometry. All esds (except the esd in the dihedral angle between two l.s. planes) are estimated using the full covariance matrix. The cell esds are taken into account individually in the estimation of esds in distances, angles and torsion angles; correlations between esds in cell parameters are only used when they are defined by crystal symmetry. An approximate (isotropic) treatment of cell esds is used for estimating esds involving l.s. planes.
Refinement. Refinement of *F*^2^ against ALL reflections. The weighted *R*-factor wR and goodness of fit *S* are based on *F*^2^, conventional *R*-factors *R* are based on *F*, with *F* set to zero for negative *F*^2^. The threshold expression of *F*^2^ > σ(*F*^2^) is used only for calculating *R*-factors(gt) etc. and is not relevant to the choice of reflections for refinement. *R*-factors based on *F*^2^ are statistically about twice as large as those based on *F*, and *R*- factors based on ALL data will be even larger.

### Fractional atomic coordinates and isotropic or equivalent isotropic displacement parameters (Å^2^)

**Table:**

	*x*	*y*	*z*	*U*_iso_*/*U*_eq_	
V1	0.16311 (2)	0.04276 (6)	0.66648 (3)	0.04096 (14)	
O1	0.16555 (9)	0.2270 (2)	0.57877 (11)	0.0533 (4)	
O2	0.09673 (11)	0.5226 (3)	0.48044 (13)	0.0624 (5)	
O3	0.21107 (9)	−0.1967 (2)	0.72412 (10)	0.0464 (4)	
O4	0.46575 (11)	−0.2270 (3)	0.81503 (15)	0.0860 (7)	
H4B	0.4400	−0.1379	0.7867	0.129*	
O5	0.13675 (10)	0.1611 (3)	0.74736 (12)	0.0599 (5)	
O6	0.07767 (10)	−0.0955 (3)	0.62167 (11)	0.0561 (4)	
N1	0.29210 (10)	0.0886 (3)	0.68123 (11)	0.0377 (4)	
N2	0.33889 (10)	−0.0525 (3)	0.73138 (12)	0.0411 (4)	
C1	0.21307 (13)	0.3854 (3)	0.56461 (14)	0.0432 (5)	
C2	0.29647 (12)	0.3942 (3)	0.59948 (13)	0.0390 (5)	
C3	0.34579 (14)	0.5570 (3)	0.57913 (16)	0.0487 (6)	
H3A	0.4017	0.5617	0.6015	0.058*	
C4	0.31173 (16)	0.7083 (4)	0.52658 (16)	0.0562 (6)	
H4A	0.3445	0.8155	0.5129	0.067*	
C5	0.22816 (16)	0.7015 (4)	0.49361 (16)	0.0554 (6)	
H5A	0.2052	0.8063	0.4588	0.066*	
C6	0.17849 (14)	0.5428 (4)	0.51131 (15)	0.0488 (5)	
C7	0.05967 (18)	0.6762 (5)	0.4246 (2)	0.0739 (8)	
H7A	0.0016	0.6478	0.4095	0.111*	
H7B	0.0865	0.6818	0.3722	0.111*	
H7C	0.0659	0.8027	0.4544	0.111*	
C8	0.33318 (12)	0.2369 (3)	0.65380 (14)	0.0406 (5)	
H8A	0.3903	0.2414	0.6705	0.049*	
C9	0.29058 (12)	−0.1970 (3)	0.75157 (14)	0.0397 (5)	
C10	0.32708 (14)	−0.3646 (3)	0.80484 (14)	0.0431 (5)	
C11	0.41233 (15)	−0.3729 (4)	0.83309 (17)	0.0577 (6)	
C12	0.44452 (19)	−0.5360 (5)	0.8821 (2)	0.0744 (9)	
H12A	0.5014	−0.5432	0.9001	0.089*	
C13	0.3936 (2)	−0.6854 (5)	0.90395 (19)	0.0723 (8)	
H13A	0.4162	−0.7932	0.9370	0.087*	
C14	0.3096 (2)	−0.6792 (4)	0.87791 (17)	0.0642 (7)	
H14A	0.2753	−0.7815	0.8937	0.077*	
C15	0.27640 (16)	−0.5201 (4)	0.82817 (16)	0.0508 (6)	
H15A	0.2195	−0.5164	0.8099	0.061*	
C16	0.00782 (18)	−0.1796 (5)	0.6540 (2)	0.0879 (10)	
H16A	−0.0371	−0.1897	0.6073	0.132*	
H16B	−0.0088	−0.0958	0.6997	0.132*	
H16C	0.0216	−0.3106	0.6771	0.132*	

### Atomic displacement parameters (Å^2^)

**Table:**

	*U*^11^	*U*^22^	*U*^33^	*U*^12^	*U*^13^	*U*^23^
V1	0.02696 (19)	0.0439 (2)	0.0521 (2)	−0.00458 (14)	0.00533 (14)	0.00005 (17)
O1	0.0359 (8)	0.0547 (10)	0.0670 (10)	−0.0086 (7)	−0.0028 (7)	0.0155 (9)
O2	0.0453 (9)	0.0705 (11)	0.0685 (11)	−0.0006 (8)	−0.0056 (8)	0.0233 (10)
O3	0.0364 (8)	0.0468 (9)	0.0555 (9)	−0.0077 (6)	0.0038 (6)	0.0039 (8)
O4	0.0380 (9)	0.0995 (15)	0.1183 (17)	0.0049 (10)	0.0011 (10)	0.0564 (14)
O5	0.0471 (9)	0.0652 (11)	0.0693 (11)	0.0029 (8)	0.0147 (8)	−0.0103 (9)
O6	0.0390 (8)	0.0612 (10)	0.0664 (11)	−0.0148 (7)	−0.0006 (7)	0.0052 (9)
N1	0.0290 (8)	0.0415 (9)	0.0428 (9)	−0.0003 (7)	0.0047 (7)	−0.0014 (8)
N2	0.0316 (8)	0.0416 (10)	0.0498 (10)	0.0015 (7)	0.0034 (7)	0.0016 (8)
C1	0.0384 (11)	0.0482 (12)	0.0440 (12)	−0.0035 (9)	0.0093 (9)	0.0036 (10)
C2	0.0353 (10)	0.0434 (11)	0.0395 (11)	−0.0033 (8)	0.0093 (8)	−0.0034 (10)
C3	0.0429 (12)	0.0551 (14)	0.0493 (13)	−0.0115 (10)	0.0105 (10)	0.0000 (11)
C4	0.0586 (15)	0.0576 (15)	0.0542 (14)	−0.0149 (12)	0.0146 (11)	0.0099 (13)
C5	0.0616 (15)	0.0556 (15)	0.0497 (13)	−0.0044 (12)	0.0096 (11)	0.0153 (12)
C6	0.0451 (12)	0.0575 (14)	0.0440 (12)	0.0000 (10)	0.0063 (10)	0.0059 (11)
C7	0.0635 (17)	0.083 (2)	0.0713 (18)	0.0100 (15)	−0.0089 (14)	0.0247 (17)
C8	0.0290 (9)	0.0464 (12)	0.0469 (12)	−0.0031 (8)	0.0069 (8)	−0.0045 (10)
C9	0.0355 (10)	0.0433 (11)	0.0407 (11)	0.0005 (9)	0.0067 (8)	−0.0054 (10)
C10	0.0500 (12)	0.0418 (12)	0.0392 (11)	0.0033 (9)	0.0115 (9)	−0.0041 (10)
C11	0.0471 (13)	0.0680 (16)	0.0588 (15)	0.0118 (12)	0.0101 (11)	0.0116 (14)
C12	0.0619 (17)	0.085 (2)	0.077 (2)	0.0244 (16)	0.0101 (15)	0.0246 (17)
C13	0.095 (2)	0.0673 (19)	0.0561 (16)	0.0218 (17)	0.0154 (15)	0.0142 (15)
C14	0.098 (2)	0.0459 (14)	0.0519 (15)	−0.0048 (14)	0.0225 (14)	0.0012 (13)
C15	0.0631 (15)	0.0473 (13)	0.0436 (12)	−0.0025 (11)	0.0127 (11)	−0.0072 (11)
C16	0.0597 (17)	0.101 (2)	0.103 (2)	−0.0432 (17)	0.0113 (16)	0.004 (2)

### Geometric parameters (Å, °)

**Table:**

V1—O1	1.8277 (17)	C4—H4A	0.9300
V1—O3	1.9436 (16)	C5—C6	1.377 (3)
V1—O5	1.5736 (18)	C5—H5A	0.9300
V1—O6	1.7351 (16)	C7—H7A	0.9600
V1—N1	2.0963 (17)	C7—H7B	0.9600
O1—C1	1.341 (3)	C7—H7C	0.9600
O2—C6	1.358 (3)	C8—H8A	0.9300
O2—C7	1.423 (3)	C9—C10	1.467 (3)
O3—C9	1.306 (2)	C10—C15	1.397 (3)
O4—C11	1.353 (3)	C10—C11	1.398 (3)
O4—H4B	0.8200	C11—C12	1.389 (4)
O6—C16	1.406 (3)	C12—C13	1.361 (4)
N1—C8	1.292 (3)	C12—H12A	0.9300
N1—N2	1.384 (2)	C13—C14	1.372 (4)
N2—C9	1.303 (3)	C13—H13A	0.9300
C1—C2	1.393 (3)	C14—C15	1.379 (4)
C1—C6	1.406 (3)	C14—H14A	0.9300
C2—C3	1.405 (3)	C15—H15A	0.9300
C2—C8	1.426 (3)	C16—H16A	0.9600
C3—C4	1.367 (3)	C16—H16B	0.9600
C3—H3A	0.9300	C16—H16C	0.9600
C4—C5	1.388 (3)		
			
O5—V1—O6	107.53 (9)	O2—C7—H7A	109.5
O5—V1—O1	106.03 (9)	O2—C7—H7B	109.5
O6—V1—O1	98.75 (8)	H7A—C7—H7B	109.5
O5—V1—O3	100.64 (8)	O2—C7—H7C	109.5
O6—V1—O3	89.96 (7)	H7A—C7—H7C	109.5
O1—V1—O3	147.80 (7)	H7B—C7—H7C	109.5
O5—V1—N1	101.68 (8)	N1—C8—C2	124.10 (18)
O6—V1—N1	149.00 (8)	N1—C8—H8A	118.0
O1—V1—N1	82.66 (7)	C2—C8—H8A	118.0
O3—V1—N1	74.46 (6)	N2—C9—O3	121.3 (2)
C1—O1—V1	135.54 (14)	N2—C9—C10	118.93 (19)
C6—O2—C7	117.5 (2)	O3—C9—C10	119.78 (19)
C9—O3—V1	118.22 (14)	C15—C10—C11	118.7 (2)
C11—O4—H4B	109.5	C15—C10—C9	120.0 (2)
C16—O6—V1	135.18 (19)	C11—C10—C9	121.3 (2)
C8—N1—N2	115.74 (16)	O4—C11—C12	117.9 (2)
C8—N1—V1	128.47 (14)	O4—C11—C10	122.7 (2)
N2—N1—V1	115.67 (12)	C12—C11—C10	119.5 (3)
C9—N2—N1	109.38 (16)	C13—C12—C11	120.6 (3)
O1—C1—C2	121.3 (2)	C13—C12—H12A	119.7
O1—C1—C6	119.17 (19)	C11—C12—H12A	119.7
C2—C1—C6	119.5 (2)	C12—C13—C14	121.0 (3)
C1—C2—C3	119.8 (2)	C12—C13—H13A	119.5
C1—C2—C8	120.72 (19)	C14—C13—H13A	119.5
C3—C2—C8	119.42 (19)	C13—C14—C15	119.4 (3)
C4—C3—C2	120.2 (2)	C13—C14—H14A	120.3
C4—C3—H3A	119.9	C15—C14—H14A	120.3
C2—C3—H3A	119.9	C14—C15—C10	120.8 (3)
C3—C4—C5	119.9 (2)	C14—C15—H15A	119.6
C3—C4—H4A	120.1	C10—C15—H15A	119.6
C5—C4—H4A	120.1	O6—C16—H16A	109.5
C6—C5—C4	121.4 (2)	O6—C16—H16B	109.5
C6—C5—H5A	119.3	H16A—C16—H16B	109.5
C4—C5—H5A	119.3	O6—C16—H16C	109.5
O2—C6—C5	125.1 (2)	H16A—C16—H16C	109.5
O2—C6—C1	115.6 (2)	H16B—C16—H16C	109.5
C5—C6—C1	119.2 (2)		
			
O5—V1—O1—C1	68.2 (2)	C7—O2—C6—C5	0.8 (4)
O6—V1—O1—C1	179.4 (2)	C7—O2—C6—C1	−178.6 (2)
O3—V1—O1—C1	−76.5 (3)	C4—C5—C6—O2	−178.7 (2)
N1—V1—O1—C1	−31.9 (2)	C4—C5—C6—C1	0.6 (4)
O5—V1—O3—C9	−90.59 (16)	O1—C1—C6—O2	2.2 (3)
O6—V1—O3—C9	161.58 (15)	C2—C1—C6—O2	−179.7 (2)
O1—V1—O3—C9	55.0 (2)	O1—C1—C6—C5	−177.2 (2)
N1—V1—O3—C9	8.72 (14)	C2—C1—C6—C5	0.9 (3)
O5—V1—O6—C16	−27.1 (3)	N2—N1—C8—C2	178.26 (18)
O1—V1—O6—C16	−137.1 (3)	V1—N1—C8—C2	−6.0 (3)
O3—V1—O6—C16	74.0 (3)	C1—C2—C8—N1	−6.8 (3)
N1—V1—O6—C16	132.6 (3)	C3—C2—C8—N1	174.9 (2)
O5—V1—N1—C8	−85.9 (2)	N1—N2—C9—O3	1.1 (3)
O6—V1—N1—C8	113.8 (2)	N1—N2—C9—C10	179.94 (17)
O1—V1—N1—C8	18.99 (19)	V1—O3—C9—N2	−8.7 (3)
O3—V1—N1—C8	176.1 (2)	V1—O3—C9—C10	172.45 (14)
O5—V1—N1—N2	89.78 (15)	N2—C9—C10—C15	179.3 (2)
O6—V1—N1—N2	−70.5 (2)	O3—C9—C10—C15	−1.9 (3)
O1—V1—N1—N2	−165.29 (15)	N2—C9—C10—C11	−0.8 (3)
O3—V1—N1—N2	−8.15 (13)	O3—C9—C10—C11	178.0 (2)
C8—N1—N2—C9	−177.45 (19)	C15—C10—C11—O4	−178.7 (3)
V1—N1—N2—C9	6.3 (2)	C9—C10—C11—O4	1.5 (4)
V1—O1—C1—C2	29.7 (3)	C15—C10—C11—C12	1.0 (4)
V1—O1—C1—C6	−152.29 (18)	C9—C10—C11—C12	−178.9 (2)
O1—C1—C2—C3	176.2 (2)	O4—C11—C12—C13	178.6 (3)
C6—C1—C2—C3	−1.8 (3)	C10—C11—C12—C13	−1.1 (5)
O1—C1—C2—C8	−2.0 (3)	C11—C12—C13—C14	0.3 (5)
C6—C1—C2—C8	180.0 (2)	C12—C13—C14—C15	0.6 (4)
C1—C2—C3—C4	1.2 (3)	C13—C14—C15—C10	−0.7 (4)
C8—C2—C3—C4	179.5 (2)	C11—C10—C15—C14	−0.1 (3)
C2—C3—C4—C5	0.3 (4)	C9—C10—C15—C14	179.7 (2)
C3—C4—C5—C6	−1.2 (4)		

### Hydrogen-bond geometry (Å, °)

**Table:**

*D*—H···*A*	*D*—H	H···*A*	*D*···*A*	*D*—H···*A*
O4—H4B···N2	0.82	1.84	2.568 (2)	147
C8—H8A···O4^i^	0.93	2.32	3.243 (3)	169

Symmetry codes: (i) −*x*+1, *y*+1/2, −*z*+3/2.
